# Cardiotoxicity of anti-cancer drugs: cellular mechanisms and clinical implications

**DOI:** 10.3389/fcvm.2023.1150569

**Published:** 2023-09-08

**Authors:** Cecilia Kwok, Mark Nolan

**Affiliations:** ^1^ Department of Medicine, Western Health, Melbourne, VIC, Australia; ^2^ Department of Medicine, Peter MacCallum Cancer Centre, Melbourne, VIC, Australia; ^3^ Cardiovascular Imaging, Baker Heart and Diabetes Institute, Melbourne, VIC, Australia

**Keywords:** pathophysiology, cardiotoxicity, chemotherapy, anti-neoplastic agent, anti-cancer (anticancer) medications, cardio-oncology

## Abstract

Cardio-oncology is an emerging field that seeks to enhance quality of life and longevity of cancer survivors. It is pertinent for clinicians to understand the cellular mechanisms of prescribed therapies, as this contributes to robust understanding of complex treatments and off-target effects, improved communication with patients, and guides long term care with the goal to minimise or prevent cardiovascular complications. Our aim is to review the cellular mechanisms of cardiotoxicity involved in commonly used anti-cancer treatments and identify gaps in literature and strategies to mitigate cardiotoxicity effects and guide future research endeavours.

## 1. Introduction

There is increasing survivorship of patients with cancer due to improvements in early cancer detection and treatments (1–3). While cardiovascular disease is a leading cause of morbidity and mortality worldwide (4), the added metabolic stress and adverse effects of anti-cancer drugs can lead to detrimental effects on the cardiovascular system (5). Consequences of chest radiotherapy, chemotherapy and immunotherapy can manifest as cardiomyopathy, vascular disease including hypertension, thromboembolism, conduction abnormalities, and metabolic disorders that are collectively described under the term, cancer therapy-related cardiovascular toxicity (6).

Cardio-oncology is an emerging field that seeks to improve quality of life and survival of many survivors of cancer. Improved clinician understanding of mechanisms of prescribed therapies is pertinent, as it can contribute to increased accuracy of and empowering communication with patients. Explaining to patients the basic science behind pathologies and maladies promote patient engagement and active participation in complex treatment decision-making (7). Clinical research and drug development informed by understanding of cellular mechanisms will be less likely to produce off-target effects, which makes understanding the biological basis of anti-cancer therapies critical for clinical researchers in cardio-oncology (8).

An better understanding of the cellular mechanisms behind chemotherapy-related cardiotoxicity could assist with appropriate use of imaging and biomarkers to monitor myocardial damage, guide long term cardiac care, with the goal to ultimately minimise or prevent cardiovascular complications. Thus, our aim is to review the cellular mechanisms of cardiotoxicity involved in commonly used anti-cancer treatments. We hope to identify gaps in literature and strategies to mitigate the effects of cardiotoxicity that could guide future research endeavours.

## 2. Anthracyclines

**Figure 1:**
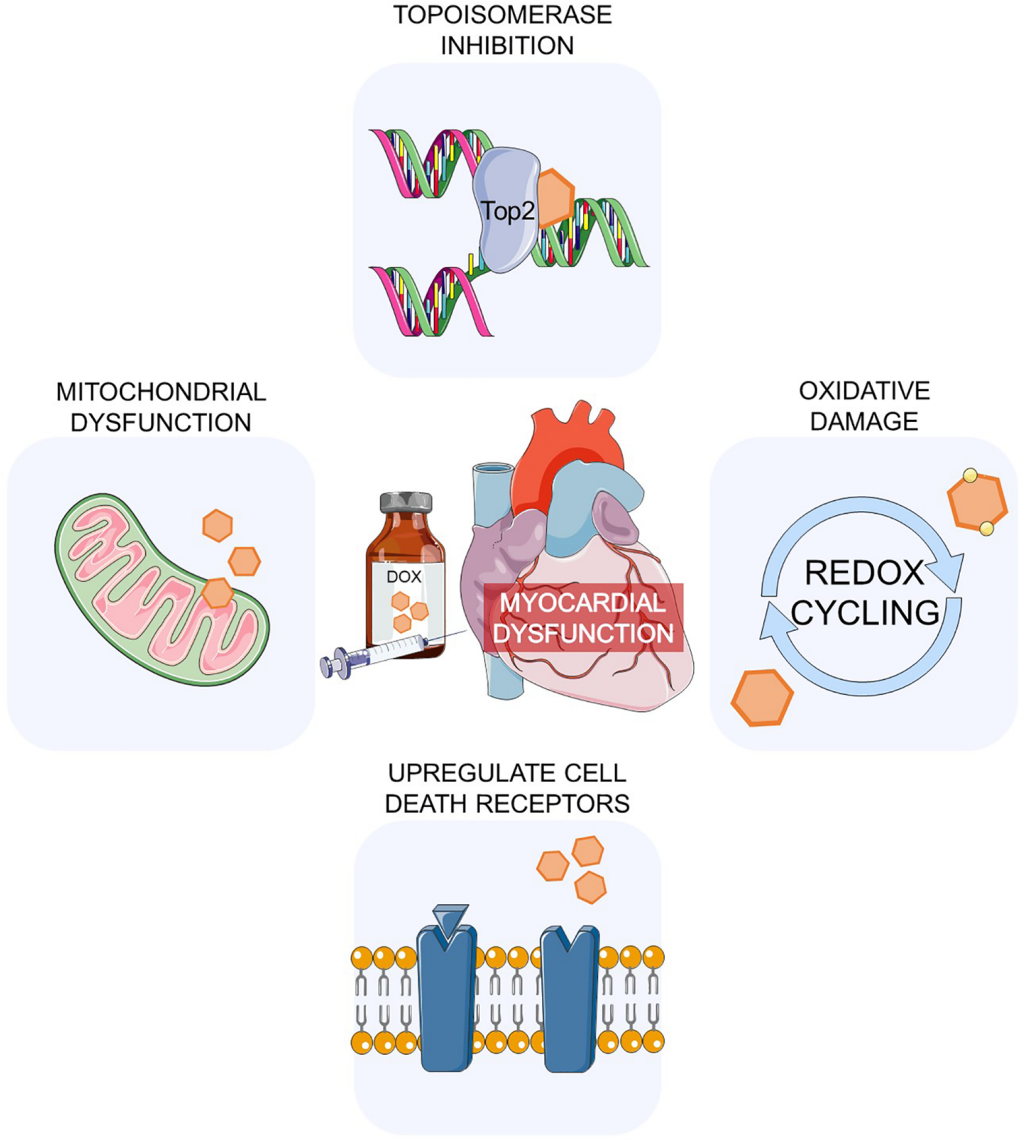
Mechanisms of doxorubicin-induced myocardial dysfunction. Doxorubicin inhibits DNA replication by intercalating with DNA and stabilising Topoisomerase 2 cleavage complex, triggering its degradation and intrinsic apoptosis. Doxorubicin's quinone structure is prone to free radical generation leading to increased generation of reactive oxygen species. Proteins of the electron transport chain in the mitochondria undergo conformational changes that result in loss of mitochondrial membrane potential, loss of ATP production, and activation of casepase-3 mediated apoptosis. Various cell death receptors in cardiomyocytes have been shown to be upregulated including proteins pivotal in apoptosis, such as autophagy, RIPK1/3-dependent necroptosis, ferroptosis, and pyroptosis. The net effect is decreased cardiomyocyte contractility and cell death, resulting in fibrosis and continuous compensatory hypertrophic remodelling of remaining cardiomyocytes. The Figure was partly generated using Servier Medical Art, provided by Servier, licensed under a Creative Commons Attribution 3.0 unported license.

Anthracycline agents (e.g., doxorubicin, daunorubicin, epirubicin, idarubicin) remain highly effective and are first-line treatment options for haematological cancers, breast cancers and sarcomas in both adults and children (9, 10), however, recent prescribing patterns suggest a fall in anthracycline use (11). Anthracycline-induced cardiotoxicity (ACT) is a final by product of iterative myocardial damage that begins at time of first exposure and may trigger a continuous remodelling process, though this may not manifest clinically until decades. Clinically, ACT can be characterized by the time of presentation into three categories: acute, early-onset progressive, or late-onset progressive (10). Acute manifestations of toxicity may include sinus tachycardia, arrhythmias or atrioventricular block. Early-onset progressive disease presents within a year of treatment completion, with subclinical findings of reduced left ventricular (LV) fractional shortening, loss of myocyte contractility and increased afterload that is indicative of abnormal LV function. Late-onset progressive disease present more than a year after treatment with increased afterload, LV wall thinning, dilated cardiomyopathy with symptoms of congestive heart failure. Genetic variation of multiple genes associated with anthracycline-induced cardiotoxicity play a significant role in determining individual sensitivity to ACT, in both adult and paediatric populations, with certain genes also impacted in other oncological mechanisms of cardiotoxicity (7). This adds to the complexity to identifying the exact mechanism of ACT, although several theories have been proposed—(i) topoisomerase-2 inhibition (12–14), (ii) reactive oxygen species (ROS) generation (15), (iii) mitochondrial dysfunction leading to reduced adenosine triphosphate (ATP) production (16–18), and (iv) effects on other programmed cell death pathways (Figure 1) (19, 20). As the mechanism of doxorubicin has been extensively studied, it will be the focus of this discussion.

Topoisomerases are intracellular enzymes and work as part of the DNA replication machinery by catalysing breaks in DNA and then repairing these breaks (21). Anthracyclines have the ability to inhibit topoisomerase activity and hence prevent cellular proliferation or repair. There are two major forms of topoisomerase-2 (Top2); Top2α is only expressed in proliferating cells and is widely regarded as the main target of doxorubicin's anti-cancer effects, while Top2β is present in all mammalian cells including cardiomyocytes. Due to their sequence similarity, doxorubicin binds non-selectively to both isoforms. Doxorubicin intercalates with DNA, stabilises Top2 cleavage complex and blocks DNA replication fork progression (22). As the replication machinery fails to proceed with DNA replication, the cleavage complex triggers proteasomal degradation of Top2β, which exposes double stranded DNA breaks and initiate a cascade of p53-mediated events, such as mitochondrial dysfunction and intrinsic apoptosis (22–24). This is consistent with findings that Top2β deletion in murine embryonic fibroblasts and cardiomyocytes protect against doxorubicin-induced DNA damage, ROS accumulation, and apoptosis (14, 25). p53 deletion attenuates doxorubicin-induced cardiotoxicity in mice (26).

Oxidative damage from ROS accumulation was one of the first proposed mechanisms of doxorubicin-induced cardiotoxicity. It's based upon doxorubicin's ability to undergo redox cycling, and mainly attributed to its quinone structure that is prone to free radical generation leading to increased levels of ROS (15). There are higher levels of ROS generating enzymes such as NADPH oxidase 2 (Nox2) (27) and increased levels of transcriptional antioxidant proteins via activation of nuclear factor erythroid 2-related factor 2 (Nrf2), a key factor in cardioprotection from doxorubicin (28). Cardiomyocytes are thought to be highly susceptible to free radical damage due to an abundance of mitochondrial electron transport chain (ETC), a prominent location for ROS formation (29). However, this theory has been challenged in recent years by lack of evidence of redox cycling in cell lines treated with doxorubicin (30), and this level of oxidative damage was not able to be replicated in an *in vivo* rat model of doxorubicin-induced cardiotoxicity (29, 31). Further, oxidative stress can make the myocardium more sensitive to pro-arrhythmic effects of drugs that block potassium channels, reducing their repolarisation reserve, thereby increasing their vulnerability to drugs that trigger Torsades de pointes (32).

The link between increased ROS and onset of cellular senescence is firmly established (33, 34). Senescence is characterized as a cellular stress response that can elicit cell reactions resulting in temporary adaptations to stressors, induce autophagy, or activate cell death (35). Senescent cells play a role in the aging process by not only entering a state of cell cycle arrest but also by manifesting a senescence-associated secretory phenotype. Cellular senescence has been observed in cardiac fibroblasts (36), endothelial cells (37), and myocytes (38), and others have proposed a link between the accumulation of senescent cells in the heart and the primary cause of cardiovascular complications that manifest in patients undergoing anthracycline chemotherapy (39). Doxorubicin is known to induce cells into senescence though the mechanism is yet to be fully elucidated (39).

Doxorubicin downregulates nuclear factors important in cardiac metabolism and promotes mitochondrial biogenesis, such as peroxisome proliferator-activated receptor gamma co-activator 1α (PPARPGC1α) and co-activator 1B (PPARPGC1β). Their scarcity contribute to increased ROS generation and apoptosis (40). Transcriptional analysis of mouse cardiac muscle treated with doxorubicin or DMNQ, a redox-cycling agent with similar redox properties, revealed that mitochondrial ETC proteins underwent conformational changes that led to loss of mitochondrial membrane potential, loss of ATP production, and activation of caspase-3 mediated apoptosis (41). Doxorubicin has also been shown to play a role in upregulating inflammatory factors such as interleukin-1 (IL-1) and tumour necrosis factor-α (TNF-α) in the myocardium, which can trigger immune responses (42). Additionally, doxorubicin can activate cell death receptors in cardiomyocytes (19) leading to further immune activation. Other cell death pathways, such as RIPK1/3-dependent necroptosis (43), abnormal iron homeostasis leading to ferroptosis (38), and nucleotide-binding domain, leucine-rich-containing family, NOD-like receptor family pyrin domain-containing 3 (NLRP) inflammasome activation resulting in pyroptosis have all been implicated in doxorubicin-induced cardiotoxicity (20, 44). The net effect is decreased cardiomyocyte contractility and cell death, eventuating in fibrosis and continuous compensatory hypertrophic remodelling of remaining cardiomyocytes (45). The clinical consequences of doxorubicin, which acts through the various mechanisms outlined, is extensive. Acutely, it can cause arrhythmias and hypotension, while in the long term, doxorubicin can contribute to cardiac hypertrophy and manifest as symptoms of heart failure (46).

Dexrazoxane is the only approved pharmacological treatment for doxorubicin-induced cardiotoxicity, however recent studies suggest that it does not completely mitigate all effects (47). Metabolites of dexrazoxane are effective iron chelators that prevent free oxygen radical formation, which supports the ROS theory (48). Interestingly, other iron chelators (e.g., deferoxamine, deferiprone, deferasirox) have only demonstrated variable cardioprotective effects against doxorubicin in various cell and animal models of cardiotoxicity (49–51). Potentially dexrazoxane may work by stabilising Top2 before doxorubicin is able to form the cleavage complex (52). Other potential novel cardioprotective strategies such as using liposomal-encapsulated anthracyclines (53), requires further research. Navitoclax, an orally active Bcl-2 inhibitor, shows promise as a senolytic agent specifically targeting senescent cells (54). Cellular senescence and oxidative and stress remain possible targets for treatment of ACT and are an active area of research with specific targets still under investigation. Future cardioprotective drug development of anthracycline alternatives that selectively inhibit Top2β may prove to be less cardiotoxic (14).

## 3. Anti-HER2 monoclonal antibodies

Currently, there are three anti-human epidermal growth factor receptor (HER2) monoclonal antibodies approved for use--trastuzumab, pertuzumab, and margetuximab. Trastuzumab is a humanised monoclonal antibody that target the extracellular domain of HER2, a member of the erythroblastic leukemia viral oncogene homolog 2 (ErbB2) family of transmembrane receptor tyrosine kinases. Twenty percent of all breast and gastric cancers are HER2 over-expressing (55, 56), and trastuzumab is the recommended first line treatment. Clinically, trastuzumab treatment alone may cause reduced LV ejection fraction but typically do not cause arrhythmias. However, cardiotoxic effects are potentiated when used in combination with anthracyclines or other chemotherapies, such as in the case of metastatic HER2 positive breast cancers (57). Trastuzumab must be used sequentially rather than concomitantly with anthracyclines along with careful consideration of background cardiac risk factors and individualised cardiac surveillance is recommended (57).

**Figure 2:**
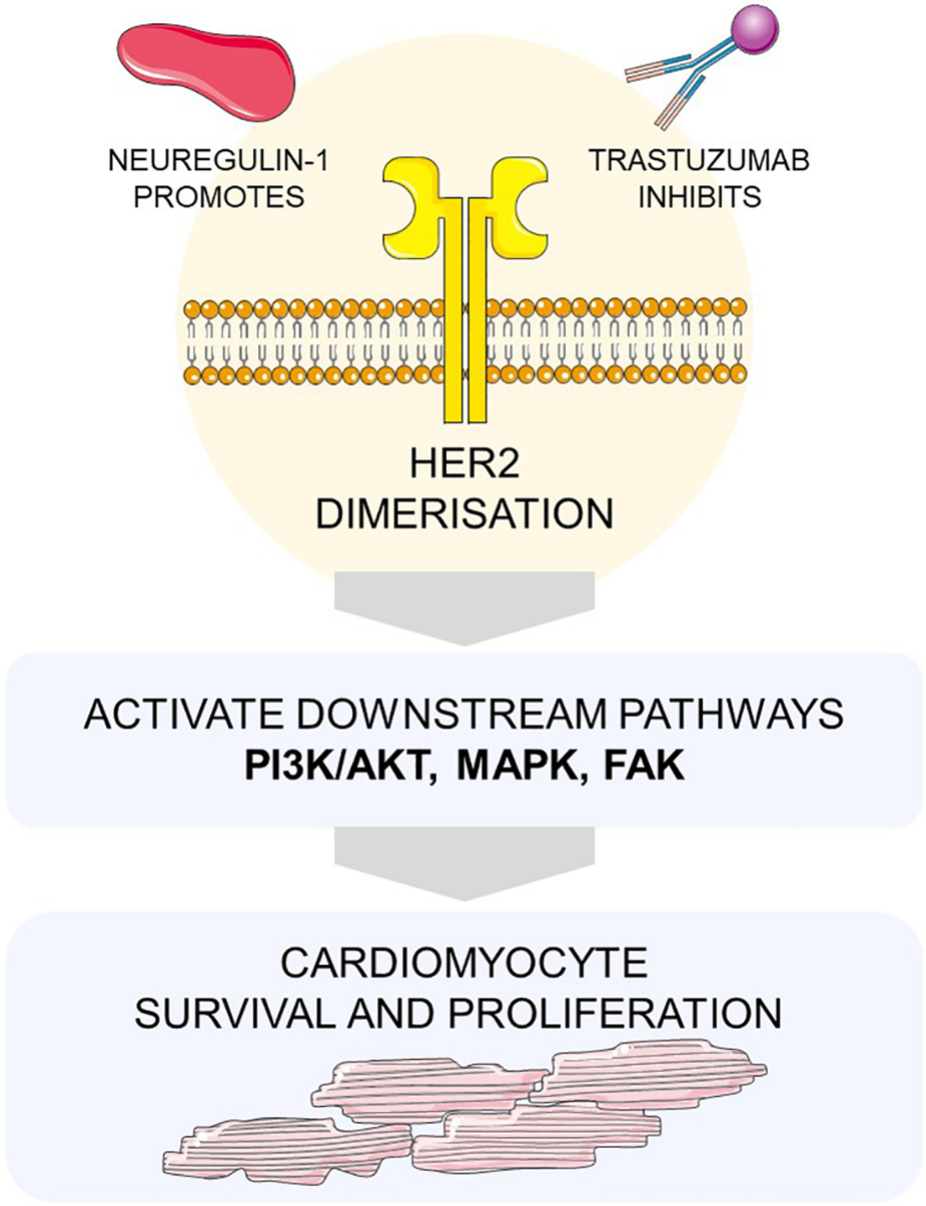
Trastuzumab inhibits HER2 receptor activation Overexpression of human epidermal growth factor receptor 2 (HER2) in malignant cells result in uncontrolled downstream activating effects of three major growth signalling pathways of phosphoinositide 3-kinases (PI3K), Akt, mitogen-activating kinase (MAPK), and Focal Adhesion Kinases (FAK) that regulate cell proliferation, differentiation, and survival. Neuregulin-1 is a growth factor released from myocardial cells that stimulate HER2, promoting cell survival. Trastuzumab binds to HER2 and inhibit uncontrolled cell growth by HER2 receptor downregulation and cell death. The Figure was partly generated using Servier Medical Art, provided by Servier, licensed under a Creative Commons Attribution 3.0 unported license.

HER2 activation can also occur independently without specific ligand binding, leading to homo and hetero-dimerization and autophosphorylation of tyrosine kinase residues on its cytoplasmic domain. Overexpression HER2 cancer cells lead to uncontrolled activation of three major downstream growth signalling pathways—phosphoinositide 3-kinases (PI3K)/AKt (58), mitogen-activating kinase (MAPK) (59) and Focal Adhesion Kinases (FAK) (60) that regulate cell proliferation, differentiation, and survival, carefully coordinated by a complex network of intracellular feedback loops (Figure 2). Trastuzumab binding to HER2 inhibits uncontrolled cell growth by HER2 receptor downregulation, and enhances apoptosis via mitochondrial ROS and increased pro-apoptotic protein transcription (61). It also triggers antibody-dependent cell-mediated cytotoxicity—increased antigen presentation by cancer cells via upregulation of MHC molecules (62), as well as increased activation of dendritic cells (63), natural killer cells (64), and macrophages (65).

Cardiomyocytes also express members of the ErbB2 family of proteins (HER1, HER2, and HER4), and ErbB2 knock out mice develop dilated cardiomyopathy (66). Neuregulin-1 (NRG1) is a growth factor released from myocardial endothelial cells that induces HER2/HER4 heterodimerisation or HER4/HER4 homodimerization which activates major survival pathways of PI3K/AKt, MAPK/Erk, and FAK (67).

The MAPK/Erk signalling pathway is driven by numerous G protein coupled receptors, receptor tyrosine kinases, and integrins during times of myocardial stress. The precise activity of the MAPK/Erk pathway in cardiomyocyte homeostasis has been reviewed by Gilbert et al. (68). FAK is an intracellular tyrosine kinase that, when autophosphorylated, mediates signal transduction from integrins, growth factors and cytokine receptors (69) and is also a key scaffolding protein. Its central role in signal transduction and activating downstream cell proliferation and survival make it an essential oncoprotein in breast cancer (69). In a highly regulated cooperative series of phosphorylation events and effector protein recruitment, all three signalling cascades ultimately converge into cardioprotective mechanisms particularly important during times of oxidative stress (70, 71). Hence, trastuzumab interferes with these pathways and contribute to increased cardiomyocyte damage from ROS accumulation and mitochondrial dysfunction, proteasomal degradation, and foetal sarcomeric gene expression.

Trastuzumab increases oxidative stress and associated caspase 3/7 activity suggestive of apoptosis in a model of murine cardiomyocytes (72). Receptor-mediated cell death occurs via upregulation of Bcl-2 family (73). Thus, HER2 blockade by Trastuzumab affects HER2 ability to dimerise with HER4, inhibitory effects on downstream cell survivla cascade, result in ROS accumulation, mitochondrial dysregulation and upregulation of proapoptotic factors that lead to apoptosis (74, 75).

Another cell death pathway, autophagy, is also affected by trastuzumab binding. A process negatively regulated by mammalian target of rapamycin (mTOR) in nutrient rich conditions, it is the main process of protein recycling and is important for myocardium remodelling and cardiac development (76). mTOR supresses autophagy by disrupting Ulk1 and AMP-activated protein kinase (AMPK) interaction during nutrient-rich conditions and is at least partially regulated by the upstream MAPK/Erk (77). Trastuzumab blockade of HER2 signalling result in autophagy dysregulation by mTOR activation, resulting in inhibition of key autophagosome proteins, increased ROS generation and subsequent cardiotoxicity (77, 78). Compensatory mechanisms such as cardiac hypertrophy can result in autophagy inhibition.

Calcium/calmodulin-dependent protein kinase 2 (CaMK2) is a serine/threonine protein kinase that regulates cardiac excitation-contraction coupling, calcium homeostasis, and activate pathways of hypertrophy and apoptosis (79, 80). The main isoform expressed in the heart is CaMK2δ and is an attractive therapeutic target as dysregulated calcium homeostasis and excitation-contraction coupling are key contributors to arrhythmia and heart failure (79). The CaMK2 inhibitor AS105 is able to suppress calcium leak from the sarcoplasmic reticulum, preserving calcium homeostasis and suppressing arrhythmia (81). The development of CAMK2 inhibitors is challenging due to the risk of off-target effects due to its multifunctionality. For example, the splice variants of CaMK2 (e.g., CaMK2δB and CaMK2δC) localise to the nucleus compared with cytoplasm, and can exert opposing cardioprotective and proapoptotic effects respectively (80). The two isoforms also respond differently to calcium homeostasis and transcriptional regulation (82). β-adrenergic receptor antagonists are effective treatment of trastuzumab-related cardiomyopathy as β-adrenergic receptor stimulation result in disrupted calcium homeostasis in a CaMK2-dependent manner (83).

## 4. Bruton tyrosine kinase inhibitors

Ibrutinib, a first-generation oral small molecule, is a selective inhibitor of Bruton's Tyrosine Kinase (BTK), a critical component of the B cell receptor (BCR) pathway in B cell lymphomas. Due to its efficacy, it was rapidly approved for first line treatment of chronic lymphocytic leukaemia and is now also used for mantle cell lymphoma, Waldenström's macroglobulinemia and marginal zone lymphoma (84, 85).

The BCR is composed of immunoglobulin heavy and light chains, enabling it to recognize a wide array of unique antigens pivotal for B cell growth, development, and maturation via four main signaling cascades—MAPK, nuclear factor kappa-light-chain-enhancer of activated B cells (NF-кB), Akt, and mTOR—all of which play crucial roles in cell survival (86). Downstream in this process, BTK remains consistently active in B cell lymphomas. Ibrutinib functions by covalently binding to a cysteine residue within the active site of BTK, resulting in irreversible inhibition of this pathway.

It is well established that ibrutinib is independently associated with developing atrial fibrillation (AF) (87–89), with an estimated cumulative incidence at two years after treatment of 10.3% (90). AF is associated with significant morbidity, as it can lead to heart failure, stroke and death. Ibrutinib also exhibits interactions with agents commonly used to treat AF, such as diltiazem, verapamil, digoxin, and amiodarone, via the cytochrome 3A4/5 pathways (91). Further, ibrutinib is associated with increased risk of bleeding (92), which complicates anticoagulation therapy. Supraventricular tachycardia, nonsustained ventricular tachycardia (93), heart failure, hypertension (94, 95), conduction disorders (96) and Takotsubo cardiomyopathy have also been reported following ibrutinib treatment (97).

C-terminal SRC kinase (CSK) is an inactivator of the inflammation-associated Src kinases by phosphorylation of its C-terminus (98). Ibrutinib binds reversibly to CSK, leading to Src-related inflammation. A study by Xiao et al found a cardiomyocyte-specific CSK knock out mouse, high serum inflammatory markers (TNF-α and IL-6), increased apoptotic cells, macrophage/monocytic infiltration, and left atrial fibrosis were found in the cardiomyocyte-specific CSK knock out mouse compared with wild type, which they postulated was possibly related to the IL-6/JAK/STAT pathways (99). They also found higher incidence of AF, left atrial enlargement, fibrosis, and elevated CaMK2 in ibrutinib-treated mice compared with wild type, that was not replicated in a BTK knock out mouse model or mice treated with a more specific BTK inhibitor, acalabrutinib. This supports the hypothesis that ibrutinib causes AF via off-target effects by binding to other kinases that contain similar cysteine residues, which is consistent with clinical reports (100). It was suggested that left atrial inflammation and fibrosis leading to AF may be due to persistently reduced CSK levels when ibrutinib is used long term (99).

CaMK2-dependent phosphorylation of the ryanodine receptor 2 in cardiomyocyte may impair intracellular calcium homeostasis and abnormal atrial electrical conductance, which may be linked with higher AF inducibility, increased left atrial mass and fibrosis (12). Others reported that CaMK2 can induce NLRP3 inflammasome (101), and while NLRP3 inflammasome has been linked with AF (102), the direct effects of CSK and CaMK2 have yet to be elucidated (103).

Other potential mechanisms of ibrutinib-induced cardiotoxicity under investigation are the BTK-mediated cardioprotective PI3K/Akt pathway and increased ROS production. Neonatal rat ventricular myocytes treated with ibrutinib were found to have reduced PI3K/Akt levels (104), while an increase in PI3K activity resulted in less atrial fibrosis and cardiac conduction (105). Mice with reduced PI3K/Akt activity were found to be more susceptible to AF (105) and had increased ROS production which promoted CaMK2 activation in mice treated with ibrutinib, a likely contributor to atrial fibrosis, remodelling and AF (106). These effects were ablated by apocynin, an inhibitor of NADPH oxidase that resulted in less oxidative stress.

Ibrutinib has the propensity to bind to other enzymatic structures similar to BTK that contain the target cysteine residue, such as IL-2-inducible T cell kinase (ITK), tyrosine kinase expressed in hepatocellular carcinoma (TEC), and hematopoietic cell kinase (HCK), resulting in off-target effects (107). Furthermore, plasma analysis from patients treated with ibrutinib revealed six plasma biomarkers related to cardiovascular diseases, which may be representative of some of the off-target effects observed. Estupiñán et al. generated a novel knock in mouse (serine substituted for cysteine 481 in the BTK active site) resistant to ibrutinib binding could be useful in future studies to further elucidate molecular mechanisms of ibrutinib-induced cardiotoxicity (108). Finally, ibrutinib may exert its hypertensive effects by downstream VEGF inhibition leading to reduced nitric oxide and increased production of endothelin-1, resulting in increased vascular tone (109, 110).

## 5. 5-flurouracil-based agents

5-fluorouracil (5-FU) and its oral prodrug, capcitabine, is a synthetic pyrimidine antimetabolite commonly used for breast, head and neck, and gastrointestinal malignancies. Its anti-tumour effects mainly occur during the S phase of the cell cycle by inhibiting thymidylate synthase (TS), which is encoded by the gene *TYMS* and incorporating its active metabolites into RNA and DNA, causing genomic instability. Patients with pre-existing CVD or risk factors of CVD, concurrent or history of chest radiotherapy, or administration of other cardiotoxic drugs are more likely to develop 5-FU cardiotoxicity. Furthermore, variants in the genes encoding TS and dihydropyrimidine dehydrogenase (DPYD), the main enzyme in fluorouracil catabolism, are associated with cardiotoxicity (111–114). Administration schedule with continuous infusion compared with bolus infusion is also associated with more severe cardiac events (115, 116). Effects of 5-FU cardiotoxicity appear reversible upon drug withdrawal (117, 118). The most well-studied mechanisms are vasospasm likely secondary to endothelial and smooth muscle cell (SMC) dysfunction and thrombosis.

Coronary vasospasm clinically presents similar to ACS with chest pain, dyspnoea, palpitations, blood pressure variation and can be accompanied with serum troponin rise and ECG features of ST segment changes (119), however with normal findings on coronary angiography (120). Coronary vasospasm is mediated by vasoconstrictor peptides like endothelin-1 and protein kinase C leading to increased vascular tone (121, 122), and studies have noted its dose-dependent effects are effectively terminated with nitrates and calcium channel blockers (123, 124).

5-FU has effects on reduced levels of protein C and increased fibrinopeptide A (122, 125), which promotes a hypercoagulable environment. In addition to endothelial dysfunction, these effects trigger the clotting cascade of platelet and fibrin accumulation and lead to thrombus formation. In addition, myocardial ischaemia can result from erythrocyte membrane changes that lead to increased blood viscosity and decreased oxygen carrying capacity (126). Other processes such as direct myocardial damage from apoptosis and ferroptosis (127, 128), ROS accumulation and autophagy (129), Krebs cycle dysfunction (130), and vacuolization of sarcoplasmic reticulum (131) have also been reported.

## 6. Vascular endothelial growth factor antagonists

Vascular Endothelial Growth Factor (VEGF) is a signaling protein that promotes angiogenesis with additional positive effects on endothelial function, cardiac contractility, and vasodilation (132). VEGF is produced in nearly all vascularized tissue by endothelial cells, fibroblasts, macrophages and platelets and production is particularly high in fenestrated and sinusoidal blood vessels. VEGF antagonists have been demonstrated to be efficacious in several cancers, including colorectal cancer, renal cancer, non-small-cell lung cancer and gastric cancer.

Adverse cardiovascular effects of VEGF inhibitors include hypertension, QTc prolongation, LV systolic dysfunction and nephrotic syndrome. Approximately 80%–90% of patients will have a rise in their blood pressure with VEGF inhibitors (133) and an estimated 25% will develop hypertension requiring treatment (134). The odds of clinical hypertension are increased more than five-fold in patients with cancer treated with VEGF Inhibitors. (134) The pathophysiology of hypertension due to VEGF inhibitors include; (i) reduced nitric oxide (NO) production by endothelial cells which can trigger immune cell infiltration and vascular inflammation causing damage to the endothelium (135); (ii) increased endothelin-1 production by endothelial cells (136); and (iii) capillary rarefaction (137). VEGF inhibitors enhances the activity of intracellular protein C, leading to the activation of the Akt/PKB pathway. This fosters the growth of endothelial cells and processes that enhance survival during hypoxia, including fatty acid oxidation, glycolysis, and improved mitochondrial homeostasis (138, 139). The mechanisms by which VEGF inhibitors increase endothelin-1 levels is currently unknown.

VEGF is likely essential for healthy renal glomerular function and VEGF inhibitors may also cause renal dysfunction. Knockout mice with VEGF-A gene deletion demonstrated loss of podocyte foot processes and endothelial fenestrations and perturbations of renal glomerular protein barrier. Human podocyte-specific VEGF-A deletion in adult kidneys similarly result in kidney injury (140). The mechanisms of which are likely due to reducing local NO production and by inhibiting nephrin production by renal podocytes via the Akt/PKB pathway (141). These intracellular actions have the cumulative downstream effects of abnormal glomerular barrier function and proteinuria. This pathophysiology shares similarities with that of pre-eclampsia, where the soluble VEGF-A receptor, Flt-1, is produced in excess and traps circulating VEGF rendering it inactive (142).

VEGF inhibitors are associated with increased risk of both venous and arterial thromboembolic events (143). This is likely secondary to reduced endothelial NO production, as NO down-regulates IL-1 induced expression of leukocyte adhesion molecules, and thereby prevents inflammatory cell recruitment and proliferation within the endothelium (144). Endogenous VEGF inhibitors have a role in activating platelets as NO reduces function of the platelet thromboxane A2 receptor thereby increases local coagulability (145).

## 7. Small molecule tyrosine kinase inhibitors

Constituting 15% of newly diagnosed leukemia cases, chronic myeloid leukemia (CML) arises due to a reciprocal translocation between chromosomes 9 and 22. This translocation results in the formation of the BCR-ABL fusion oncogene, which fuels the proliferation of cells (146). Novel small-molecule tyrosine-kinase inhibitors (TKI) directed specifically against the BCR-ABL gene has changed CML from a cancer with high mortality to largely a chronic disease managed with medications (147). However cardiovascular toxicity is an increasing concern with each progressive generation of TKI agents.

The first TKI developed for CML was imatinib, which was initially very effective. However, over 30% of patients would develop resistance over time; as the kinase domain pocket that interacts with imatinib was vulnerable to disruption by large number of mutations that did not affect oncogenic properties. Second-generation TKI agents nilotinib and dasatinib were developed as alternatives to imatinib for patients with resistance. Cardiovascular adverse effect profiles for these agents differ, suggesting possible role of off-target effects as these TKIs can inhibit over 60 different intracellular kinases with differing specificity profiles for each agent (148).

Nilotinib is associated with higher rates of clinically significant peripheral arterial disease compared to imatinib (36% vs. 6%) (150). The mechanism was proposed to be due to increased serum levels of leukocyte adherence molecules, VCAM and ICAM-1. It is possible that nilotinib induces dose-dependent downregulation of adipogenic regulatory genes, such as peroxisome proliferator-activated receptor-alpha, *PPARA* and *LPIN1*, which increases endothelial inflammation and local levels of oxidized low-density lipoprotein (151). Nilotinib has not been associated with LV dysfunction (143) or observed to demonstrate any effect on *in vitro* foetal rat cardiomyocytes (152). Nilotinib is associated with increased QTc prolongation and higher risk of ventricular arrythmias (153). The pro-arrhythmic mechanism has yet to be identified and raises the intriguing possibility that tyrosine kinases may be involved in the natural anti-arrhythmic protection of cardiomyocytes.

Dasatinib is a second-generation TKI that is active in most imatinib-resistant CML cases. It is complicated by pleural effusion in approximately 30% of patients (143), and high numbers of natural killer cells in pleural fluid suggest an inflammatory aetiology (153). Pulmonary arterial hypertension occurs in about 0.5% of dasatinib-treated patients (154), and can occasionally be progressive despite withdrawal of treatment. It is possible that this may be due to dasatinib inhibition of two kinases of the Src family protein tyrosine kinase that are highly expressed in human pulmonary artery smooth muscle cells (155).

Ponatinib, a third-generation TKI treatment for CML, contrasts the first and second generation TKIs due to its efficacy against the T315Z mutation variant of BCR-ABL oncogene (156). Ponatinib has been associated with higher rates of cardiovascular side-effects than first and second generation TKIs, with approximately 26% of ponatinib-treated patients developing cardiovascular complications in the PACE study at 3 years (157). The most common adverse effects were arterial and venous thromboembolic events. Around 25% of individuals experienced deteriorating hypertension, and approximately 5% encountered heart failure as well (158). It is likely that cardiomyopathic effects are mediated by interference with cellular pro-survival pathways as ponatinib inhibits Akt/Erk kinases which increases intracellular caspase-3-induced apoptosis (159). Pre-treating cell-cultures with neuregulin prevented ponatinib-induced apoptosis, providing further support for cellular survival pathways in ponatinib-cardiotoxicity. Furthermore, ponatinib affects endotheilal survival and reduces angiogenesis by inhibiting the Notch-1 pathway (160), and may also increase circulating levels of von Willebrand factor due to endothelial damage, which may explain high incidence of arterial thromboembolism (161).

## 8. Proteasome inhibitors

Proteasome inhibitors (PI) are considered standard maintenance therapy for multiple myeloma and have been demonstrated to increase 5-year survival from 25% to 52% (162). They block the 26S proteasome of the ubiquitin-proteasome system, which normally identifies proteins marked for degradation. Proteasome inhibition causes transport failure of undesirable proteins to lysosomes leading to increased intracellular accumulation and apoptosis. As multiple myeloma cells produce greater amounts of potentially toxic proteins than non-cancer cells, myeloma cells are especially vulnerable to proteasomal inhibition (143).

Bortezomib is a first-generation proteasome inhibitor and is first-line treatment for multiple myeloma. It is associated with clinical hypotension and hypertension (6) but is considered to have less cardiotoxicity than other PIs (163). Bortezomib is associated with higher rates of peripheral neuropathy than other PIs due to off-target effects on intra-cellular serine proteases (164).

Carfilzomib, an irreversible PI used for relapsed or refractory multiple myeloma, has been demonstrated to cause a 31% reduction in disease progression when added to the standard regimen of lenalidomide and dexamethasone (165). Its use is associated with high incidence of adverse cardiovascular side-effects. An analysis of four prospective studies found 22% incidence of adverse cardiovascular events, including 13% incidence of arrhythmia, 7% incidence of heart failure, and 14% incidence of clinical hypertension (166). Carfilzomib is also associated with increased risk of AF, venous thromboembolism, hypertension (165) as well as QTc prolongation and pericardial effusion (166). Cardiotoxicity risk appears to be reversible with cessation of treatment (167).

The wide spectrum of manifestations of carfilzomib cardiotoxicity possibly suggests multiple pathophysiologies, including increased vascular oxidative stress, endothelial dysfunction and inhibition of endothelial proliferation. Carfilzomib has been shown to enhance the function of intracellular enzymes, specifically serine-threonine protein phosphatase 2A. This, in turn, leads to the inhibition of the AMPKα/mTORC1 pathway, a key player in the down-regulation of autophagy-related proteins (168), which has been associated with the development of left ventricular dysfunction (169). Interestingly, metformin is an activator of AMPKα pathway and *in vitro* experiments suggest it could prevent carfilzomib cardiotoxicity without impairing proteasomal inhibition (170). Further studies are needed to determine if this approach has clinical utility.

Carfilzomib may also cause endothelial dysfunction by inhibiting the PI3K/Akt pathway which is required for nitric oxide synthase activation, leading to lower levels of endothelial NO production (170). This is supported by human studies which have demonstrated impaired endothelial function after carfilzomib administration (171, 172). Carfilzomib-related endothelial dysfunction may also increase risk of myocardial ischemia as it has been observed to cause coronary vasoconstriction in rabbit models (173).

## 9. Chimeric antigen receptor T-cell therapy

Chimeric Antigen Receptor T-cell Therapy (CAR-T) is a novel therapy for relapsed or refractory leukemias or aggressive lymphomas (174). Described as a “living drug,” CAR-T involves extracting a patient's T-cells from their plasma. These T-cells are subsequently subjected to bench-side re-engineering using a viral vector, a process where artificial proteins known as chimeric antigen receptors are introduced onto the surface of the T-cells and are designed to identify cancer antigens. The modified T-cells are then reintroduced into the patient's bloodstream.

Cardiotoxicity from CAR-T usually occurs in the context of a generalized cytokine release syndrome (CRS) or immune-mediated myocarditis (175). CRS, which cause excessive release of pro-inflammatory cytokines leading to systemic and cardiac inflammation, occurs in 60%–90% of treated patients depending on CAR-T product and tumour burden (176) and has a variable presentation in terms of both symptoms and severity with common presenting symptoms including fever, tachypnoea, tachycardia, hypotension or hypoxia. CRS is graded on a scale from 1 (mild) to 4 (life-threatening). Activation of re-introduced T-cells can cause cascade activation of further inflammatory cells including T-helper cells, B-cells and macrophages, leading to high serum levels of inflammatory cytokines such as interleukin-2 (IL-2), interleukin-6 (IL-6), interferon-γ (IFN-γ) (177). These proinflammatory cytokines can generate oxidative stress, mitochondrial dysfunction and altered intracellular calcium cycling which may culminate in myocardial ischemia and impaired myocardial contractility (178). Elevated inflammatory proteins activate the vascular endothelium to release further cytokines, creating a positive CRS feedback loop (179). Further downstream complications of this inflammatory cascade can include capillary leakage and consumptive coagulopathy in severe cases.

Cardiotoxicity presentations can range from asymptomatic myocardial biomarker elevation to severe heart failure. A prospective single-centre registry of 137 patients treated with CAR-T for haematological malignancy found that 54% developed a detectable rise in troponin and 28% had a significant drop in their left ventricular ejection fraction (LVEF), defined as an absolute LVEF decrement >10% to <50% (180). There was a 12% incidence of severe cardiovascular events including cardiovascular death, clinical heart failure or arrhythmia. For patients with a serious cardiovascular clinical event, 95% were preceded by a detectable troponin elevation, suggesting that serial troponin measurements could be a viable monitoring strategy, and the risk of events was reduced by early administration of tocilizumab, a monoclonal antibody against IL-6 receptor.

## 10. Immune checkpoint inhibitors

Immune Checkpoint Inhibitors (ICI) are a novel class of therapeutics designed to boost anti-cancer effects of the native immune system and are associated with substantial improvements in overall and progression-free survival in a number of malignancies (181). It has been estimated that ICIs can be beneficial in over 60 different cancer types and that approximately 43% of all cancer patients may be eligible for ICI therapy (182).

The activation of T-cells in response to threats such as microbes or cancerous cells necessitates a connection between a T-cell and an antigen-presenting cell (APC). T-cell activation is initiated by foreign peptides showcased on APCs coupled with the major histocompatibility complex (MHC). The co-stimulatory signal is conveyed through the APC ligand CD80, which triggers cell activation and subsequent proliferation. To prevent autoimmunity against self-antigens, T-cells have inhibitory ligands such as cytotoxic T-lymphocyte-associated protein-4 (CTLA-4) or programmed-cell-death-protein-1 (PD-1) that compete for binding to APC ligand CD80 which result in T-cell anergy by inhibiting intracellular signaling. These immunosuppressive ligands act as “checkpoints” that can allow cancer cells to escape immunological surveillance. Immune checkpoint inhibitors such as monoclonal antibodies against CTLA-4, PD-1 and other inhibitory ligands, promote T cell activation and anti-cancer effect, which is partially offset by increased autoimmune side-effects.

ICI are associated with a spectrum of cardiotoxicity presentations, of which the most severe is fulminant myocarditis. Although rare with reported incidence of 0.5%–1.7% (183, 184), ICI myocarditis is associated with mortality rates of 35%–50%. Majority of cases present early with ∼76% myocarditis cases presenting within 6 weeks of ICI administration and overall median time to onset of 27 days. Exact mechanism of ICI myocarditis is unknown but possibilities include shared antigen between cardiomyocytes and immune-mediated upregulation of pre-existing autoantibodies, myocardial metabolism dysregulation by inflammatory cytokines or response to CRS (185).

Supporting the hypothesis of T cell activation against shared or homologous myocardial antigens, T cell infiltrate has been observed in myocardial biopsies of fulminant ICI myocarditis (186). Inflammatory cytokines such as IFN-γ increases expression of programmed death-ligand (PD-L1) in cardiomyocytes and might potentially play a protective role for the heart, as evidenced by the development of premature dilated cardiomyopathy in PD-1 knockout mice (187, 188). The potential involvement of PD-L1 can be supported by animal studies that reveal T-cell infiltration into the myocardium, accompanied by the presence of detectable PD-1 and PD-L1 molecules (189).

ICI agents are also associated with three-fold increased risk of myocardial infarction in large patient registries (190). Potential mechanisms include increased caspase production by activated T-cells, subsequently increasing intra-plaque production of IL-6 and TNF-α. This cascade results in macrophage activation and the destabilization of caps on atherosclerotic plaques. Further support for this pathophysiology was an imaging substudy of 40 ICI-treated patients patients that reported a three-fold increase in the rate of total atherosclerotic plaque progression (190). An autopsy study of 11 patients treated with ICIs identified an inflammatory infiltrate primarily composed of lymphocytes within coronary arteries, which contrasts with the usual macrophage-pre-dominant infiltrate observed in atherosclerotic plaques (191).

Pericardial disease also account for 0.4% of adverse effects attributed to ICI agents in pharmacovigilance studies while pericardial disease has been reported to four-fold higher in ICI-treated patients than in controls (192). Case reports of Takotsubo cardiomyopathy secondary to ICI exposure have also been published (193).

## 11. Summary

**Figure 3:**
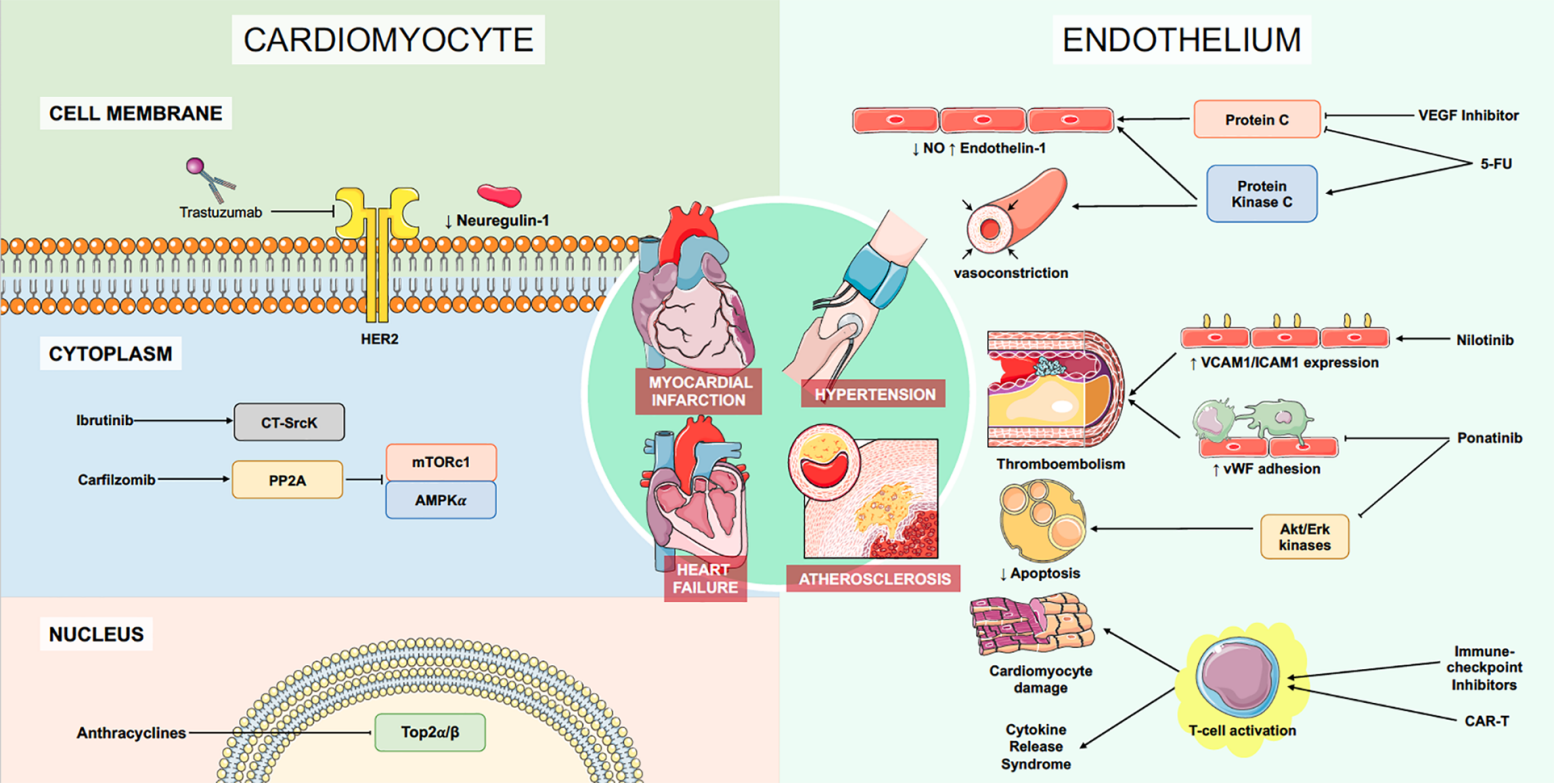
Overall summary of molecular mechanisms of cardiotoxicity for commonly-used agent classes. 5-FU, 5-Flurouracil; AMPK, adenosine monophosphate-activated protein kinase; CAR-T, chimeric antigen receptor T-cell therapy; Erk, extracellular signal-regulated kinase; HER2, human epidermal growth factor receptor 2; ICAM1, Intercellular adhesion molecule-1; mTORC1, mammalian target of rapamycin complex 1; NO, nitric oxide; PP2A, Protein phosphatase 2A; Top2, Topoisomerase-2; VEGF, vascular endothelial growth factor; vCAM1, vascular cell adhesion molecule-1; vWF, von Willebrand factor. Figure was partly generated using Servier Medical Art, provided by Servier, licensed under a Creative Commons Attribution 3.0 unported license.

Recent advances in cancer treatments have translated into substantial improvements in patient survival and quality of life. These treatments have become increasingly better targeted to specific intracellular and extracellular pathways and as a result, these pathways are now increasingly recognized to play a role in cardiomyocyte function and survival (Figure 3 and Table 1). It is now essential for the practicing cardio-oncologist to understand these molecular pathways to predict and identify phenotypes of chemotherapy-related cardiotoxicity, both in existing and investigational agents. With the advent of future generations of anti-cancer drugs, the spectrum of cardiovascular complications from anti-cancer treatments is likely to expand.

**Table 1:** Mechanism of action and cardiotoxicity for anti-cancer agents.

Anti-cancer agents	Example of agent class	Mechanism of anti-cancer action	Mechanism of cardiotoxicity	Cardiovascular complications
Anthracyclines	Doxorubicin	Topisomerase-2β Inhibition	Reactive oxygen species production	Cardiomyopathy and heart failure
Anti-HER2 monoclonal antibodies	Trastuzumab	HER2 Inhibition	Decrease in Neuregulin production	Cardiomyopathy and heart failure
Bruton tyrosine kinase inhibitors	Ibrutinib	Bruton's Tyrosine Kinase Inhibition	C-terminal Src kinase inhibition	Atrial fibrillation, pericardial effusion ventricular arrythmias
5-Flurouracil-based agents	5-flurouracil	Thymidylate Synthase Inhibition	Protein Kinase C activation and Endothelin-1 production	Coronary vasospasm
Vascular endothelial growth factor antagonists	Bevacizumab	Inhibits Vascular Endothelial Growth Factor	Decreased Endothelial NO Production	Hypertension, cardiomyopathy, arterial or venous thromboembolism, proteinuria
Small molecule TKI for CML	Ponatinib	Inhibits Intracellular Tyrosine Kinase Inhibitors involved in Cellular Proliferation	Likely off-target effects on similar intra-cellular kinases, such as Akt/ERK kinases.	Arterial or venous thromboembolism hypertension, pleural effusion, pulmonary hypertension
Proteosome inhibitors	Carfilzomib	Inhibits Proteosomal Degradation of Intracellular Proteins	Increases autophagy by Inhibiting the AMPKα/mTORC1 intracellular pathway	Arrhythmia, heart failure, hypertension
CAR-T agents	Tisagenlecleucel	T-cells re-engineered benchside to recognize cancer antigens	Cytokine release syndrome	Cardiomyopathy, myocardial Injury arrhythmias
Immune-checkpoint inhibitors	Nivolumab	Block T-cell self-regulatory pathways and increase anti-cancer activity	Likely attack self-antigens present in cardiomyocytes	Myocarditis, ischemic heart disease, pericardial disease Takot-subo's cardiomyopathy

CML, chromic myeloid leukemia; ERK, extracellular signal-regulated kinase; HER2, human epidermal growth factor recepetor-2; NO, nitric oxide; TKI, tyrosine kinase inhibitor.
